# Long-Term Cellulose Enrichment Selects for Highly Cellulolytic Consortia and Competition for Public Goods

**DOI:** 10.1128/msystems.01519-21

**Published:** 2022-03-08

**Authors:** Gina R. Lewin, Nicole M. Davis, Bradon R. McDonald, Adam J. Book, Marc G. Chevrette, Steven Suh, Ardina Boll, Cameron R. Currie

**Affiliations:** a Department of Energy Great Lakes Bioenergy Research Center, University of Wisconsin—Madison, Madison, Wisconsin, USA; b Department of Bacteriology, University of Wisconsin—Madison, Madison, Wisconsin, USA; c Wisconsin Institute for Discovery and Department of Plant Pathology, University of Wisconsin—Madison, Madison, Wisconsin, USA; California State University, Northridge

**Keywords:** *Atta colombica*, cellulose degradation, leaf-cutter ant refuse dump, microbial interactions, serial enrichment, metagenomics, metatranscriptomics

## Abstract

The complexity of microbial communities hinders our understanding of how microbial diversity and microbe-microbe interactions impact community functions. Here, using six independent communities originating from the refuse dumps of leaf-cutter ants and enriched using the plant polymer cellulose as the sole source of carbon, we examine how changes in bacterial diversity and interactions impact plant biomass decomposition. Over up to 60 serial transfers (∼8 months) using Whatman cellulose filter paper, cellulolytic ability increased and then stabilized in four enrichment lines and was variable in two lines. Bacterial community characterization using 16S rRNA gene amplicon sequencing showed community succession differed between the highly cellulolytic enrichment lines and those that had slower and more variable cellulose degradation rates. Metagenomic and metatranscriptomic analyses revealed that *Cellvibrio* and/or *Cellulomonas* dominated each enrichment line and produced the majority of cellulase enzymes, while diverse taxa were retained within these communities over the duration of transfers. Interestingly, the less cellulolytic communities had a higher diversity of organisms competing for the cellulose breakdown product cellobiose, suggesting that cheating slowed cellulose degradation. In addition, we found competitive exclusion as an important factor shaping all of the communities, with a negative correlation of *Cellvibrio* and *Cellulomonas* abundance within individual enrichment lines and the expression of genes associated with the production of secondary metabolites, toxins, and other antagonistic compounds. Our results provide insights into how microbial diversity and competition affect the stability and function of cellulose-degrading communities.

**IMPORTANCE** Microbial communities are a key driver of the carbon cycle through the breakdown of complex polysaccharides in diverse environments including soil, marine systems, and the mammalian gut. However, due to the complexity of these communities, the species-species interactions that impact community structure and ultimately shape the rate of decomposition are difficult to define. Here, we performed serial enrichment on cellulose using communities inoculated from leaf-cutter ant refuse dumps, a cellulose-rich environment. By concurrently tracking cellulolytic ability and community composition and through metagenomic and metatranscriptomic sequencing, we analyzed the ecological dynamics of the enrichment lines. Our data suggest that antagonism is prevalent in these communities and that competition for soluble sugars may slow degradation and lead to community instability. Together, these results help reveal the relationships between competition and polysaccharide decomposition, with implications in diverse areas ranging from microbial community ecology to cellulosic biofuels production.

## INTRODUCTION

Across diverse environments, especially in soil, leaf litter, and the guts of herbivores, microbes decompose recalcitrant plant biomass into energy-rich sugars. Only select microbes have the full suite of enzymes necessary to break down plant biomass, but this activity fuels complex microbial communities and helps drive the terrestrial carbon cycle (1–4). Despite the importance of plant biomass degradation to global energy and nutrient cycles, the ecological dynamics of microbial communities degrading plant biomass are poorly understood. Species diversity has a complex, variable relationship with the rate of decomposition, and the microbe-microbe interactions such as cooperation and antagonism that likely shape the ability of a community to degrade plant biomass are difficult to decipher (5–13).

While detailed studies have made progress toward understanding the community dynamics of decomposition in host-associated environments (14–17), the high diversity of plant biomass-degrading communities in soil and leaf litter often prohibits a molecular characterization of decomposition and the associated interactions. Experimental enrichments on selective substrates have proven to be a useful technique to dissect complex microbial communities (18–21). By repeatedly transferring natural communities on plant biomass or its constituent polymers, it is possible to lower the diversity of communities and identify organisms responsible for specific functions (18, 22). These studies, along with extensive characterizations of model laboratory microbes, indicate that a suite of secreted enzymes break down the polymers within the plant cell wall (2). Cellulose, a crystal of β-1,4-linked glucose molecules and the most abundant component of the plant cell wall, is catabolized extracellularly into soluble oligosaccharides, such as cellobiose, through the combined action of cellulases. Endocellulases and lytic polysaccharide monooxygenases (LPMOs) internally cleave cellulose, while exocellulases processively release cellobiose from the ends of cellulose chains (2). Cellobiose is then imported into cells, where it is catabolized by a β-glucosidase enzyme into glucose. In addition to the enzymology of cellulose degradation, studies have also suggested that interactions can play an important role in this process. It has been shown that organisms can cooperate to degrade plant biomass, together producing the different enzyme types necessary to break down cellulose into cellobiose (23, 24). In contrast, soluble cellobiose is a public good, and many noncellulolytic microbes encode β-glucosidases and can compete with cellulase producers for this sugar (21, 25, 26).

**FIG 1:**
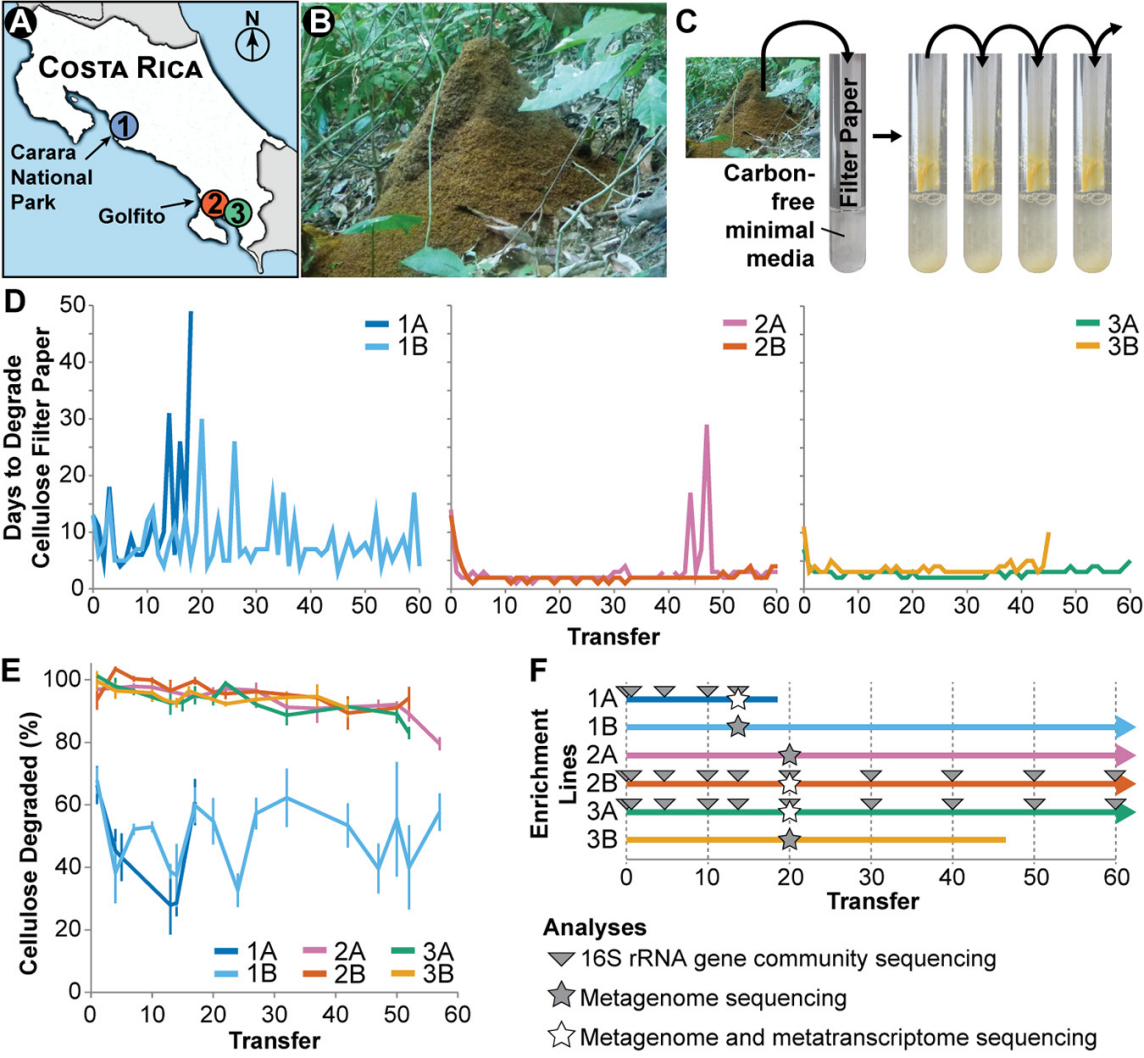
Cellulose enrichment methods and long-term experiment results. (A) Locations of leaf-cutter ant refuse dumps 1, 2, and 3. (B) Leaf-cutter ant refuse dumps are large piles of cellulose-enriched leaf material. (C) Refuse material was inoculated into test tubes with a strip of cellulose filter paper as the sole carbon source. Once the filter paper broke in half (always at the air-liquid interface), the microbial community was transferred to a fresh filter paper test tube. (D) Ability of six enrichment lines (two independent inoculations from each refuse dump) to degrade cellulose across transfers. See Fig. S1 at https://doi.org/10.6084/m9.figshare.12967751 for replicates of each enrichment line. (E) Percentage of cellulose degradation in 10 days. The amount of cellulose degraded in 10 days was quantified relative to controls using an acid detergent method with three biological replicates per sample. Error bars are standard deviation. (F) Sampling points for long-term enrichment experiment sequencing analyses.

Here, we used leaf-cutter ant refuse dumps as inoculum for experimental enrichments. In neotropical forest and savannah ecosystems, a significant percentage of plant material is degraded in refuse dumps of the dominant herbivore, leaf-cutter ants (Fig. 1A and B). Refuse dumps are created by ants as they discard cellulose- and lignin-enriched leaf material that has already been partially degraded by the ants’ fungal cultivar (27–29). Refuse dumps have a complex, highly cellulolytic microbial community (30–34). However, metagenomic analyses have not been able to discern the contributions of putative cellulolytic bacteria or their interactions with other microbes due to the community’s high diversity (31).

In this study, we examine microbe-microbe interactions that impact cellulose degradation using communities obtained from leaf-cutter ant refuse dumps. To dissect cellulose-degrading communities from this taxonomically diverse environment, we enriched six independent communities from refuse dumps on cellulose for up to 60 transfers (∼275 generations) (Fig. 1). Using metagenomics, metatranscriptomics, and physiological analysis of isolates, we characterized the ecological and physiological dynamics of these communities. Further, we analyzed the data sets for signatures of cooperation, such as complementation of cellulase functions across cellulolytic taxa, and signatures of competition, such as use of cellobiose by noncellulolytic microbes.

## RESULTS

### Cellulose degradation rates increased across enrichments but were variable in some enrichment lines.

We tracked the ability of six independent enrichment lines inoculated from three Atta colombica leaf-cutter ant refuse dumps to degrade a strip of cellulose filter paper over 60 transfers (Fig. 1). In this enrichment scheme, the community was only transferred when it demonstrated cellulolytic activity by breaking the cellulose filter paper in half (Fig. 1C). Initially, communities each degraded the cellulose filter paper between 7 and 14 days (transfer 0) (Fig. 1D). After the first two transfers, the communities in each enrichment line were able to degrade the cellulose filter paper at least twice as fast, and in enrichment line 2A, the rate of degradation increased 7-fold. Over the subsequent transfers, the degradation rate in enrichment lines from refuse dumps 2 and 3 (2A, 2B, 3A, and 3B) stabilized, with the communities consistently breaking the filter paper between 1 and 4 days. However, the ability of enrichment line 2A to degrade cellulose slowed multiple times between transfers 40 and 50, and enrichment line 3B was stopped after transfer 46 because the community did not break the cellulose filter paper after 2 months. In contrast, enrichment line 1A and 1B communities from refuse dump 1 oscillated in their abilities to degrade cellulose over time, ranging from degradation in 4 days to 30 days, and transfer 19 of enrichment line 1A did not break apart the cellulose filter paper within 2 months and, therefore, was not continued.

Replicate cultures and quantitate cellulose degradation assays further supported the cellulolytic ability in these independent focal enrichment lines. Specifically, cellulolytic ability in each of the six enrichment lines was mirrored by two additional replicates started at the first transfer and continued until transfer 20 (see Fig. S1 at https://doi.org/10.6084/m9.figshare.12967751). The replicates of enrichment lines 1A and 1B all demonstrated variability in cellulolysis, and stable, fast degradation was observed in all replicates of enrichments lines 2A, 2B, 3A, and 3B. Additionally, 73 quantitative cellulose degradation assays from across the 60 transfers found that communities from enrichment lines 2A, 2B, 3A, and 3B degraded almost all detectable cellulose in 10 days (average, 94.5 ± 4.3% cellulose degradation), while communities from enrichment lines 1A and 1B degraded between 38% and 68% of cellulose in 10 days (average, 50.9 ± 10.8% cellulose degradation) (Fig. 1E).

### Taxonomic structure of communities shifted and then stabilized across transfers.

**FIG 2:**
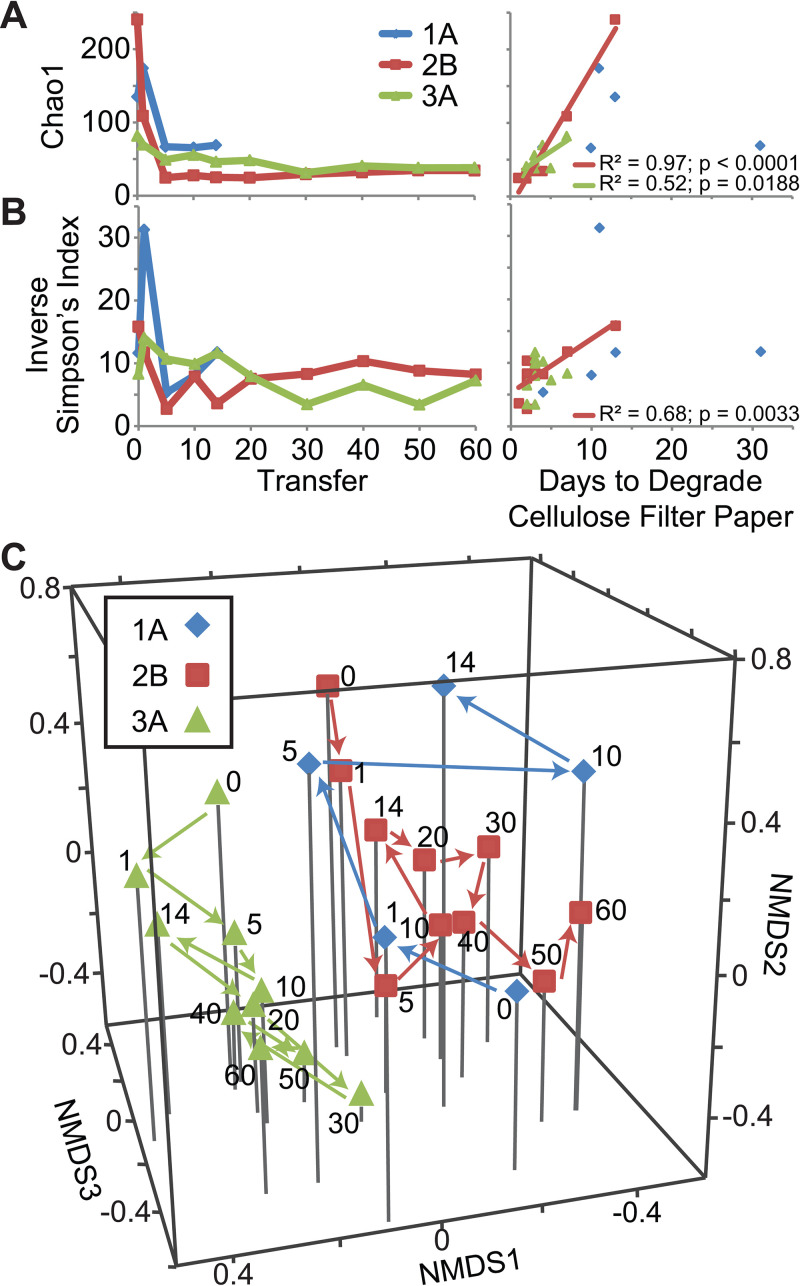
Diversity changes in enrichment lines across transfers. Shifts in Chao1 (estimated number of OTUs) (A) and inverse Simpson’s diversity index (B) across transfers and relative to the cellulolytic ability of the communities (days to degrade the cellulose filter paper). A best fit linear line is shown for the significant linear relationships between each diversity index and the days to degrade cellulose filter paper. (C) NMDS representation (stress = 0.16, *R*^2^ = 0.82) of the Bray-Curtis dissimilarity metrics between samples. The transfer number of each sample is indicated next to the corresponding point.

To examine microbial community changes associated with selection on cellulose, we analyzed the community structure for 3 enrichment lines (1A, 2B, and 3A) across transfers using 16S rRNA gene sequencing (Fig. 1F). The predicted species richness (Chao1 metric) initially decreased in all three enrichment lines then stabilized after 5 transfers (Fig. 2A), with the communities maintaining 24 to 68 predicted operational taxonomic units (OTUs), clustered at a 97% sequence similarity threshold. In addition, there was a significant positive linear correlation between the number of days to degrade the cellulose filter paper and the Chao1 species richness for lines 2B and 3A. Similarly, diversity as measured by the inverse Simpson’s index also decreased and then stabilized in all three enrichment lines, but it fluctuated more than the Chao1 index. The inverse Simpson’s index was significantly positively linearly correlated with days to degrade cellulose only for enrichment line 2B (Fig. 2B).

We further used the 16S rRNA gene sequencing data to identify changes in community structure during enrichment on cellulose. The enrichment lines significantly differed from each other (analysis of molecular variance [AMOVA], *P* ≤ 0.001 for all pairs using the Bray-Curtis dissimilarity metric). Using nonmetric multidimensional scaling (NMDS) with three dimensions (stress = 0.16; *R*^2^ = 0.82), we observed that the community from the initial test tube (transfer 0) for enrichment line 1A was distinct from that of enrichment lines 2B and 3A (Fig. 2C). Then, enrichment line 1A shifted positively along axes 2 and 3 during the first 5 transfers. In contrast, enrichment lines 2B and 3A shifted in the opposite directions, negatively along axis 2 and axis 3 during the first ∼5 transfers and then stabilized. These results mirror the trajectories of the cellulolytic ability of these lines and correspond with the finding that the number of days to degrade filter paper increased positively with axis 2 and negatively with axis 3 (Spearman correlation, *P = *0.0023 and *P = *0.0223, respectively; axis 1 not significant).

### Putative cellulolytic OTUs shifted in abundance across transfers and between enrichment lines.

**FIG 3:**
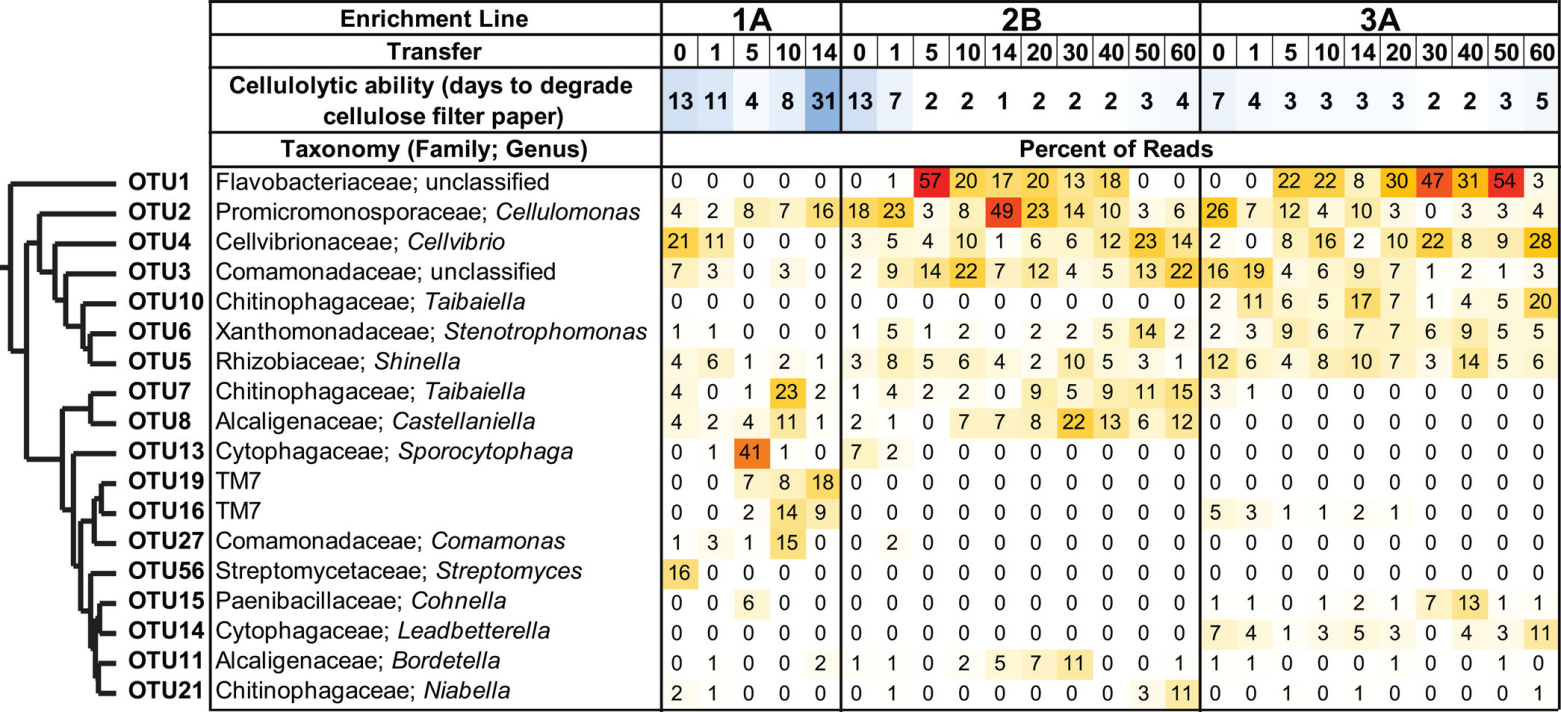
OTU patterns during long-term enrichment experiment. Heatmap showing the percentage of sequences that corresponded to each OTU. Relative abundance is shown for all OTUs that constituted at least 10% of one sample at one transfer. OTUs are clustered using Ward’s method, and their assigned taxonomy is indicated. See Fig. S2 at https://doi.org/10.6084/m9.figshare.12967751 for an expanded analysis.

Associated with the overall changes in community structure, the relative abundance of dominant OTUs varied across samples and across transfers (Fig. 3; see also Fig. S2a for an expanded version at https://doi.org/10.6084/m9.figshare.12967751). In enrichment line 1A, the most abundantly detected OTU was not consistent over time, and only a few OTUs were consistently abundant across transfers. Further, the putative cellulolytic OTU, *Cellvibrio* (OTU4), decreased in relative abundance across transfers and was replaced by a different putative cellulose degrader, *Cellulomonas* (OTU2) (35, 36). Note that this OTU2 was annotated as *Cellulosimicrobium* in the 16S rRNA gene analysis but includes the *Cellulomonas* populations identified in further analyses below (see Fig. S3 at the URL mentioned above). Thus, we will refer to this taxon as *Cellulomonas* throughout. In contrast, in the fast degradation enrichment lines (2B and 3A), sequences for *Cellulomonas* were dominant at earlier time points, while *Cellvibrio* dominated at later transfers. These shifts are also reflected in the NMDS analysis above (Fig. 2C; see also Fig. S2a at the URL mentioned above), where *Cellulomonas* (OTU2) abundance was positively correlated with axes 2 and 3 (Spearman correlation, *P = *0.00041 and *P = *0.000829, respectively), and *Cellvibrio* (OTU4) abundance was negatively correlated with axis 2 (Spearman correlation, *P = *0.000114). Further, cooccurrence network analysis demonstrated the abundances of the putative cellulose degraders *Cellulomonas* (OTU2) and *Cellvibrio* (OTU4) were negatively correlated with each other (see Fig. S2b and c at the URL mentioned above). In addition to these species-level fluctuations, clustering of the *Cellvibrio* OTU at 100% identity showed the dominant *Cellvibrio* strain shifted across transfers (see Fig. S4a at the URL mentioned above). Thus, these results indicate that the dominant putative cellulose-degrading community members do not stably coexist.

In addition, many other OTUs showed significant correlations with the NMDS analysis and in the network analysis (Fig. 2; see also Fig. S2 at https://doi.org/10.6084/m9.figshare.12967751). The negative correlation between OTU1 (unclassified *Flavobacteriaceae*) and days to cellulose degradation, as well as the strong negative correlation between this OTU’s abundance and axis 2 in the NMDS, indicates that *Flavobacteriaceae* were abundant in more cellulolytic communities. In contrast, the abundances of *Devosia* (OTU35), *Parapedobacter* (OTU59), *Cohnella* (OTU33), TM7 (OTU19), and *Taibaiella* (OTU49) were all positively correlated with days to cellulose breakdown, and the abundances of many of these OTUs were significantly positively correlated with axis 2 in the NMDS, indicating that these OTUs were found in communities that were less cellulolytic. The cooccurrence network also identified strongly positively correlated OTUs, including a cluster of positively correlated OTUs that were all found in enrichment line 1A and a second cluster of positively correlated OTUs from enrichment line 3A.

### *Cellvibrio* and *Cellulomonas* encoded and expressed overlapping sets of cellulase genes.

**FIG 4:**
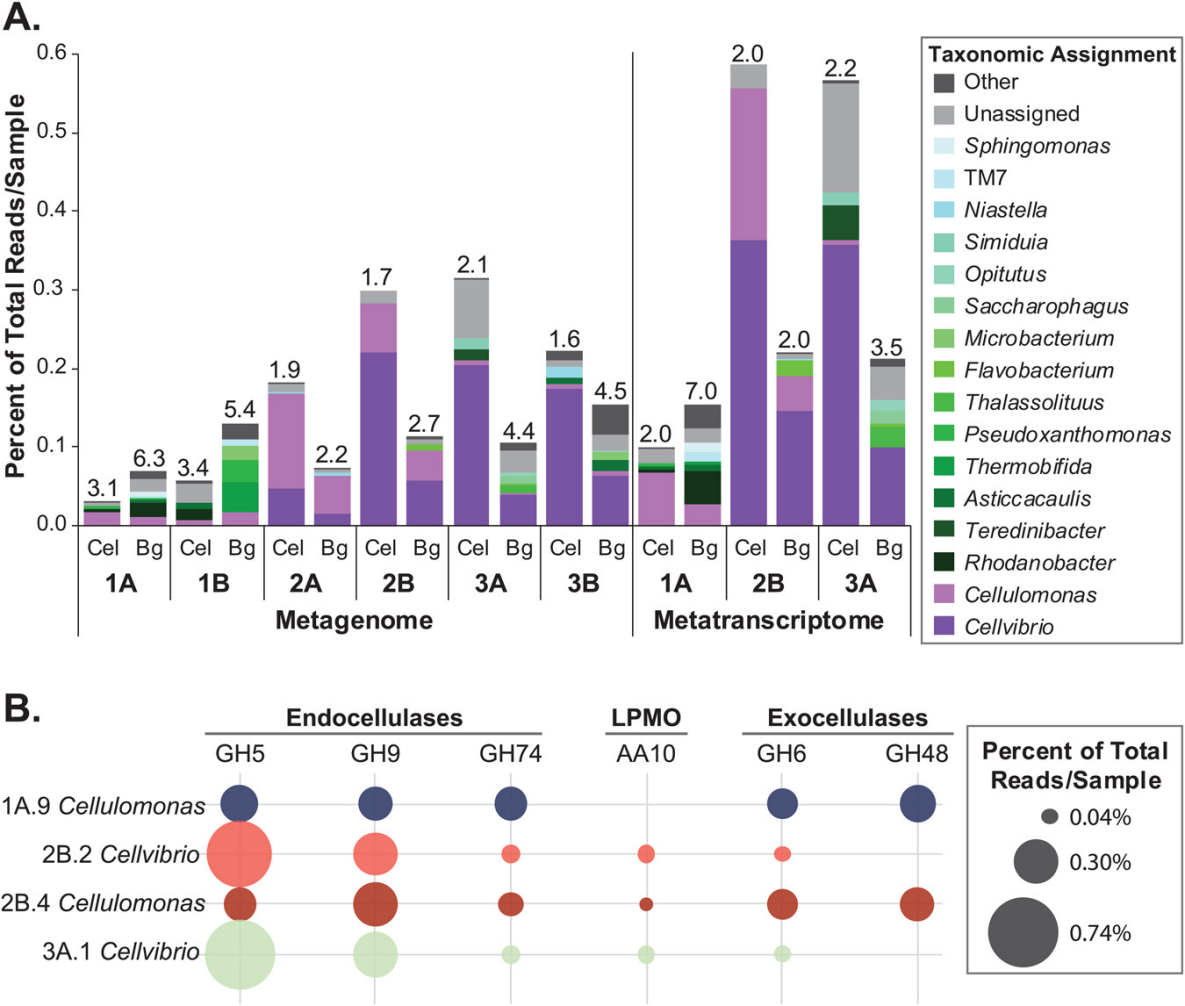
Cellulase and β-glucosidase levels in metagenomes, metatranscriptomes, and MAGs. (A) Relative levels and taxonomic assignments of cellulase (Cel) and β-glucosidase (Bg) genes and transcripts. Endocellulase, exocellulase, and LPMO CAZy classes GH5, GH6, GH7, GH9, GH12, GH48, GH74, and AA10 were included as cellulases. CAZy classes GH1 and GH3 were included as β-glucosidases. For metagenomic counts, the coverage of genes that mapped to each taxonomy was summed and normalized to the total coverage in each metagenome. For metatranscriptome counts, RNA read counts mapping to genes assigned to each taxonomy were summed and normalized to total read counts of each sample. Genera in purple (*Cellvibrio* and *Cellulomonas*) are dominant cellulose degraders across communities. The diversity (inverse Simpson’s index) of cellulase or β-glucosidase producers in each sample is indicated above the corresponding bar. (B) Relative gene expression of endocellulase, LPMO, nonreducing end exocellulase GH6, and reducing end exocellulase GH48 genes in *Cellvibrio* and *Cellulomonas* MAGs. Read counts are normalized to total mapped metatranscriptomic reads in each sample.

To confirm which organisms were degrading cellulose and understand their roles in each enrichment line, we sequenced and assembled the metagenomes and metatranscriptomes of select communities (Fig. 1F; see also Table S1 and Database S1 at https://doi.org/10.6084/m9.figshare.12967751). The assembled metagenome data sets demonstrated that enriched communities were bacteria dominated, and fungi or other eukaryote taxa were not identified. *Cellvibrio* and/or *Cellulomonas* species encoded and expressed the majority of the putative cellulases in each enrichment line, except in enrichment line 1B, where the taxonomic origin of many of the cellulase genes was unassigned (Fig. 4A). The majority of cellulase genes present and expressed in the communities coded for endocellulases from glycoside hydrolase (GH) families 5 and 9, but exocellulase and LPMO genes were also detected (see Fig. S5 at the URL mentioned above). In both the metagenomic and metatranscriptomic data, cellulase genes were over 5 times more abundant in the highly cellulolytic communities (2A, 2B, 3A, and 3B) than in the less cellulolytic communities (1A and 1B) (Fig. 4; see also Fig. S5 at the URL mentioned above).

We further investigated the contributions of *Cellvibrio* and *Cellulomonas* to cellulase production by binning the metagenomic contigs to create metagenome-assembled genomes (MAGs) (Table S2 and Database S2 at https://doi.org/10.6084/m9.figshare.12967751). MAGs for the *Cellvibrio* populations in enrichment lines 2B and 3A were estimated to be 95% and 99% complete, respectively, and MAGs for the *Cellulomonas* populations in enrichment lines 1A and 2B were estimated to be 59% and 54% complete, respectively. Both *Cellulomonas* MAGs expressed the full suite of genes necessary to deconstruct cellulose, including multiple endocellulase genes (GH families 5 and 9), a reducing end exocellulase gene (GH48), and two nonreducing end exocellulase genes (GH6) (Fig. 4B). In contrast, the *Cellvibrio* MAGs did not encode a reducing end exocellulase, but GH9 and GH5 endocellulases and an auxiliary activity (AA) family 10 LPMO were highly expressed (Fig. 4B). Further, as the *Cellulomonas* MAGs were not complete, this is likely an underestimate of their cellulolytic capacity, and additional genes were annotated as *Cellulomonas* carbohydrate-active enzyme (CAZy) genes in the metagenome (see Table S2 and Database S1 at the URL mentioned above).

To further understand the contributions of the constituent cellulase-producing organisms, we successfully isolated a *Cellvibrio* strain from enrichment line 3A at transfer 50. The 16S rRNA gene sequence of this isolate (*Cellvibrio* sp. 3A-T50a) closely matched the 16S rRNA gene sequences identified in the 3A.1 *Cellvibrio* MAG and in OTU4 in the amplicon analysis (see Fig. S4a at https://doi.org/10.6084/m9.figshare.12967751). The isolate degraded a strip of cellulose filter paper in 3 days and degraded ∼100% of available cellulose in 10 days, similar to the enrichment line that it was isolated from (see Fig. S4b and c at the URL mentioned above).

### Noncellulolytic microbes increase in abundance after filter paper degradation.

**FIG 5:**
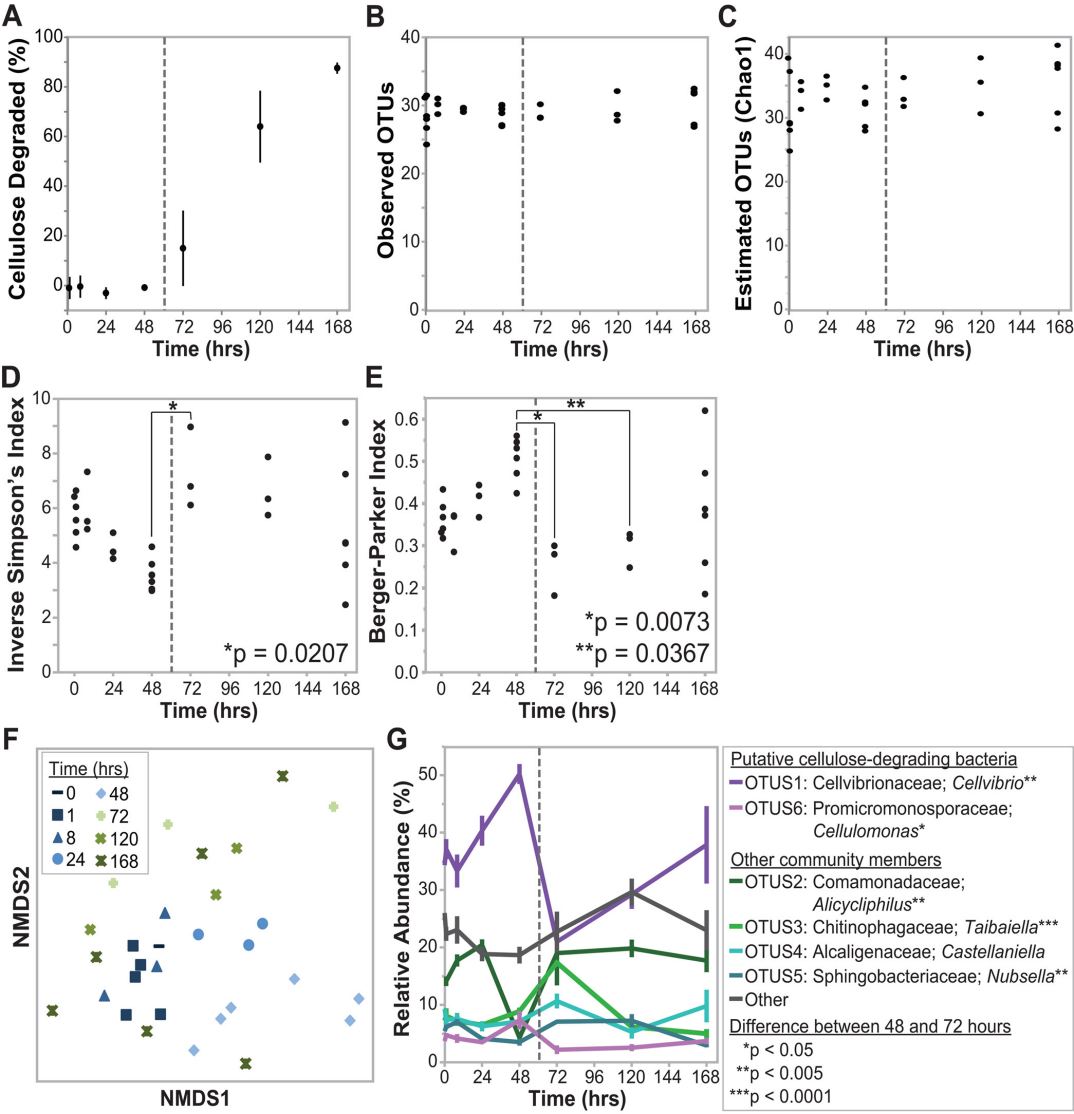
Short-term experiment. Decomposition and taxonomic shifts for enrichment line 2B across 7 days. Replicate cultures of enrichment line 2B at transfer 70 were sampled over 7 days (*n* = 3 to 6 at each time point). In all panels, the dashed line indicates when the filter paper broke in half. Diversity analyses were performed using OTUs clustered at 97% identity, and statistical analyses were performed using the Tukey-Kramer HSD test. (A) Quantitative measurement of cellulose degradation. (B) Number of observed OTUs over time. (C) Chao1 (estimated number of OTUs) over time. (D) Change in alpha diversity (inverse Simpson’s index) over time. (E) Change in Berger Parker index of dominance over time. (F) NMDS representation (stress = 0.17; *R*^2^ = 0.89) of the Bray-Curtis dissimilarity metrics between samples. (G) Change in relative abundance of the six most abundant OTUs over time.

To investigate the short-term community dynamics and interactions between the cellulolytic and noncellulolytic community members, we tracked OTU abundance over 7 days in replicate tubes of enrichment line 2B at transfer 70 (short-term experiment). These samples broke the filter paper between 48 and 72 h, and correspondingly, cellulose degradation was detected using quantitative methods starting at the 72-h time point (Fig. 5A). Of note, if this community was part of the long-term experiment, it would have been transferred at this time point, but for this specific analysis, the culture was maintained after cellulose degradation to further explore the shifts in community composition over time. Using 16S rRNA gene sequencing of this community at 0, 1, 8, 24, 48, 120, and 168 h, no significant differences were observed in the number of OTUs detected or the estimated number of OTUs (Fig. 5B and C). However, there was a significant difference overall in the inverse Simpson’s diversity index between time points (analysis of variance [ANOVA], df = 7, *F* = 2.9001, *P = *0.0263) (Fig. 5D). Specifically, we observed a >2-fold increase in this diversity index between the 48- and 72-h time points (Tukey-Kramer honest significant difference [HSD] test, *P = *0.0207), which corresponds to the interval in which the cellulose filter paper broke in half. An increase in diversity during this interval is also supported by the Berger-Parker dominance index, which differed across samples (ANOVA, df = 7, *F* = 3.4507, *P = *0.0120) and specifically decreased between the 48- and 72-h time points (Tukey-Kramer HSD test, *P = *0.0073) (Fig. 5E). NMDS representation of the Bray-Curtis dissimilarly metric also indicates a large shift in community structure between 48 h and 72 h (Fig. 5F). This shift correlates with significant decreases in the relative abundances of the putative cellulolytic *Cellvibrio* and *Cellulomonas* OTUs during this time period and significant increases in the relative abundances of the *Comamonadaceae*, *Chitinophagaceae*, and *Sphingobacteriaceae* OTUs (Fig. 5G). In sum, these data show that the community composition fluctuates within an individual culture, with a shift from cellulolytic OTUs to putative noncellulolytic OTUs and a shift to a more even community structure after initial cellulose decomposition.

### Efficient cellulolytic communities have a lower diversity of β-glucosidase-producing bacteria.

To detect organisms using the sugars released from cellulose degradation, we identified microbes that contained and expressed β-glucosidase genes, which encode enzymes to break down cellobiose into glucose (Fig. 4A). In the cellulolytic enrichment lines 2A, 2B, 3A, and 3B, over twice as many metagenomic and metatranscriptomic reads mapped to cellulase genes than β-glucosidase genes. In contrast, in enrichment lines 1A and 1B, β-glucosidase reads were approximately twice as abundant as cellulase reads. We quantified the diversity of cellulase and β-glucosidase producers in each sample using the inverse Simpson’s index (Fig. 4A). In metatranscriptomes from enrichment lines 2B and 3A, the dominant cellulase and β-glucosidase genes were assigned to *Cellvibrio* and *Cellulomonas*, and the diversity of β-glucosidase producers was at most 1.5× higher than the diversity of cellulase producers. However, in the metatranscriptome from enrichment line 1A, *Cellulomonas* dominated cellulase expression while *Rhodanobacter* was the most abundant β-glucosidase-expressing organism, and the inverse Simpson’s diversity of β-glucosidase-expressing organisms was 3.5× higher than that of cellulase-expressing organisms. Together, these findings demonstrate that a higher diversity of organisms metabolized cellobiose in the enrichment lines that were less stable and less cellulolytic.

### High expression of secondary metabolism, defensive, and localization genes indicate competition in communities.

Finally, we functionally analyzed the 25 genes with the most mapped transcripts in each of eight high-quality MAGs and found further signatures of competition. Twenty-seven of the 200 total genes analyzed are predicted to encode antagonistic rearrangement hotspot (RHS) repeat domains, toxins, and secondary metabolite production proteins (see Fig. S6a and Database S3 at https://doi.org/10.6084/m9.figshare.12967751). In particular, two separate secondary metabolism gene clusters were highly expressed in the *Cellvibrio* population 3A.1, including a hybrid type 1 polyketide synthase (PKS)-nonribosomal peptide synthase (PKS-NRPS) cluster and an NRPS cluster with high similarity to the triscatecholate siderophore turnerbactin cluster in the cellulolytic bacterium Teredinibacter turnerae T7901 (see Fig. S6b and c at the URL mentioned above) (37). Genes predicted to encode putative defensive functions were also highly expressed (see Fig. S6a at the URL mentioned above), including efflux pumps by the 1A.8 *Rhodanobacter* MAG and the 3A.7 *Opitutaceae* MAG, a gene encoding a putative penicillin binding protein by the 2B.4 *Cellulomonas* MAG, CRISPR genes, and those predicted to encode restriction enzymes. Additionally, 10% of highly expressed genes were related to localization. In all MAGs except the 3A.7 *Opitutaceae* MAG, genes involved in twitching motility (type IV pili), gliding motility, flagella-based motility, adhesion, or biofilm formation were among the 25 most highly expressed genes (see Fig. S6a at the URL mentioned above). For example, both the 2B.2 and 3A.1 *Cellvibrio* MAGs highly expressed genes predicted to be involved in twitching motility and a putative flagellar gene, and the 2B.2 *Cellvibrio* population also highly expressed two putative adhesion genes.

## DISCUSSION

Microbes are vitally important for degrading recalcitrant polysaccharides, both in association with humans (38, 39) and in the environment (1, 2, 40). Specifically, while the breakdown of plant biomass is critical for driving the global carbon cycle, the complexity of this substrate leads to intricate community dynamics and microbe-microbe interactions during decomposition (2, 13, 41, 42). In this study, we created and analyzed six independent enrichment lines of cellulose degrading communities, studying taxonomic changes over both short and long time periods and characterizing the metagenomes and metatranscriptomes of the enriched communities. Using these approaches, we found indications of competition and other negative interactions, supporting our understanding of how interactions between bacteria can structure a saprophytic community and affect its function.

### Ecological dynamics of enriched communities.

A number of studies have analyzed community succession during enrichment on plant polysaccharides (18, 20, 21, 43, 44). Here, we employed an experimental design allowing us to monitor cellulolytic ability (by observing time to break the filter paper) and community succession (through sequencing) in parallel. Overall, the enrichment approach selected for cellulolytic communities, driven by the activity of highly cellulolytic bacteria (Fig. 1 and 3; see also Fig. S4 at https://doi.org/10.6084/m9.figshare.12967751). Four enrichment lines (2A, 2B, 3A, and 3B) were relatively stable and highly cellulolytic while two enrichment lines (1A and 1B) were less stable and much less cellulolytic (Fig. 1). Our data indicate that our transfer protocols did not lead to the differences across enrichment lines because we observed high consistency in cellulolytic ability across enrichment lines from the same dump and across replicate enrichment lines (Fig. 1; see also Fig. S1 at the URL mentioned above). Instead, we hypothesize that the divergent cellulolytic and taxonomic trajectories across enrichment lines were mediated by differences in the initial community composition and by interactions between community members. Despite the enrichment lines having many—but not all—of the same initial OTUs, there are differences in the initial community in enrichment line 1A compared with the initial communities in lines 2B and 3A (Fig. 2 and 3). These differences mirror our previous study that found refuse dumps significantly vary in cellulolytic ability and microbial community composition when enriched for only one transfer (30), and inoculum source has often, but not always, been found to be a significant driver of community composition and cellulolytic ability in other systems (21, 45–48).

Despite enrichment on cellulose as the sole carbon source for ∼8 months and up to 60 serial transfers, each line maintained 24 to 68 OTUs, with some noncellulolytic OTUs constituting over 10% of the community (Fig. 2A and Fig. 3). Maintenance of richness has also been identified in other enrichment experiments both on complex biomass and microcrystalline cellulose (21, 49, 50). Our data suggest that as in previous studies (21, 51), richness was maintained by the presence of at least two trophic levels in our communities. The dominant cellulose-degrading bacteria *Cellvibrio* and *Cellulomonas* and select noncellulolytic bacteria such as the abundant OTU1 (*Flavobacteriaceae*) metabolized cellobiose through the expression of β-glucosidase genes (Fig. 4). Other community members were likely secondary consumers, as they did not encode or express cellulase genes (see Database S2 at https://doi.org/10.6084/m9.figshare.12967751). Metabolic waste products potentially could create diverse niche spaces to support many of the noncellulolytic OTUs, such as the highly abundant organisms *Comamonadaceae* (OTU3) and *Stenotrophomonas* (OTU6), which did not encode or express cellulase or β-glucosidase genes (Fig. 3 and 4).

### Signatures of competition dominate in enriched communities.

Our findings suggest antagonism was a dominant force in the communities, both among the cellulolytic species and between the cellulolytic and noncellulolytic species.

Competition for niche space between the two aerobic, cellulolytic microbes present within our communities, *Cellulomonas* and *Cellvibrio*, is supported by the negative correlation between the abundances of these organisms (Fig. 3; see also Fig. S2 at https://doi.org/10.6084/m9.figshare.12967751). While these microbes are not fully mutually exclusive, they alternate in dominance throughout the enrichment time points. Further, we did not find evidence that these microbes degraded cellulose cooperatively. Both microbes expressed a diversity of cellulose-degrading enzymes, and although the *Cellvibrio* MAGs did not encode a reducing end exocellulase gene (GH48) and thus potentially could have benefited from the *Cellulomonas* GH48, our *Cellvibrio* isolate was highly cellulolytic in monoculture (Fig. 4B, see also Fig. S4 at the URL mentioned above).

Our sequencing analyses also found competition between the cellulolytic and noncellulolytic species for the sugar cellobiose (Fig. 4A). The cellobiose-metabolizing, noncellulolytic microbes can be thought of as “cheaters”; they used the cellobiose public goods without producing or secreting cellulases, reducing cellobiose availability for the cellulolytic microbes. Previous studies have also found that cheaters use public goods during cellulose degradation and during the breakdown of other polysaccharides (21, 39, 51, 52), and cheaters have been shown to have both detrimental and positive effects on the rate of degradation (25, 53, 54). Our data suggest that in enrichment lines 1A and 1B, degradation was slowed by high levels of β-glucosidase genes from diverse microbes (Fig. 4A). Competition for cellobiose would both limit the growth of the cellulolytic microbes and promote growth of noncellulolytic microbes. In addition, we hypothesize that this abundance of β-glucosidase-producing microbes potentially contributed to the instability of these enrichment lines.

Finally, antagonistic interactions among community members were evident by the high expression of other genes related to competition (see Fig. S6 and Database S3 at https://doi.org/10.6084/m9.figshare.12967751). Examples include genes involved in RHS toxins, a polyketide-peptide, a type VI secretion system, and a siderophore. Also, the high expression of motility- and adhesion-related genes was likely important for competition for localization, as the communities were growing on a solid strip of cellulose filter paper, not in an unstructured liquid environment, and notably, biofilm production has been shown to reduce the ability of cheaters to access public goods in chitin-degrading communities (55).

While the sequencing data demonstrated that competition plays a large role in these enriched consortia, a number of positive interactions are likely also occurring. As detailed above, many microbes were likely secondary consumers and cross-fed metabolic by-products as their carbon source. Further, strong positive correlations were found between many of the OTUs, especially those in enrichment lines 1A and 3A. However, as many of these OTUs represent poorly characterized taxa, it is difficult to predict the nature of the relationships between these microbes. Additionally, we hypothesize that the *Cellulomonas* populations received amino acids and potentially other nutrients from the community, as we were unable to isolate any *Cellulomonas* strains on minimal media, and *Cellulomonas* spp. have known amino acid auxotrophies (56, 57). Potentially, on complex plant biomass, as opposed to purified cellulose, cooperation may be even more likely due to the diversity of carbon-rich polymers and other nutrients within the plant cell wall (6, 21, 58).

There are some indications that the communities that we enriched are representative of a portion of the native *Atta colombica* refuse dumps. For example, *Cellvibrio* and *Cellulomonas* are abundant in these enrichment cultures and in native refuse dumps (31). However, the conditions used for these experiments, aerobic liquid culture, are different from the refuse dump environment. These differences likely limit the diversity of microbes found in the enrichment process; for instance, we did not identify any fungi in these or similar analyses (30). Thus, there is an opportunity for future work to study the communities identified here and the broader lignocellulose-degrading communities in native refuse dumps.

### Conclusion.

We present a model in which, upon enrichment on cellulose, one or two dominant organisms are responsible for cellulose degradation, and competition for sugars is common among community members. By tracking six independent enrichment lines over time and analyzing their ecological dynamics, we identified underlying signatures of competition that link taxonomic diversity and cellulose degradation. This work provides insights into how interactions within a community influence its stability and cellulolytic ability and increases our understanding of how communities assemble and function.

## MATERIALS AND METHODS

### Refuse dump collection.

Samples were aseptically collected from the middle layer of A. colombica refuse dumps and stored at 4°C until inoculation. Material from refuse dump 1 was collected in Carara National Park, Costa Rica. Refuse dump 2 was collected at global positioning system (GPS) coordinates 8.6538°N, −83.1833°W, and refuse dump 3 was collected at GPS coordinates 8.6448°N, −83.1871°W, both in Golfito, Costa Rica (Fig. 1A). Besides originating from different *A. colombica* colonies, there were no discernible differences in the material collected. See permit information in Supplementary Methods at https://doi.org/10.6084/m9.figshare.12967751.

### Long-term experiment: enrichment procedure.

Two enrichment lines were started from each refuse dump sample (e.g., dump 2 inoculation A = enrichment line 2A). Enrichments were performed in test tubes containing 5 mL of M63 minimal medium and a 1- by 10-cm strip of Whatman Grade 1 cellulose filter paper (GE Healthcare Life Sciences, Pittsburgh, PA) pressed against the side of the tube (30). As M63 medium is defined and only contains inorganic nutrients, cellulose was the only carbon source present in each test tube, besides any trace contaminants. This environment was chosen for its ability to select for cellulolytic communities that could be used to study the ecological dynamics of cellulose degradation. While cellulolytic bacteria and fungi both grow well in this environment (59), some cellulolytic members of the dump microbial community, such as anaerobes and some fungi, are selected against by these conditions. For each enrichment line, we inoculated an approximately 3 mg (∼2-mm diameter) piece of refuse dump material into a test tube and grew the cultures shaking aerobically at 30°C. If the microbial community was capable of degrading cellulose in this test tube (referred to as transfer 0), the microbes grew directly on the cellulose filter paper and eventually broke the filter paper in half (Fig. 1C). The filter paper was confirmed not to break apart in negative control cultures, without the addition of microbes. Each tube was checked daily. After a community broke the filter paper, the test tube was vortexed, and 200 μl of culture was transferred into each of three fresh test tubes using a wide orifice p200 pipette tip. At this first transfer (transfer 1), each of these replicate tubes were run as a separate enrichment line (e.g., 2AA, 2AB, 2AC). At the second and all subsequent transfers, three replicate filter paper tubes were inoculated from each enrichment line, and the fastest tube to degrade the filter paper was transferred. After transfer 20, one of the remaining replicate lines from each original inoculation was chosen to be continued, and all other lines were discontinued (see Fig. S1 at https://doi.org/10.6084/m9.figshare.12967751). In this manuscript, we primarily focus on the initial enrichment of these communities, up to 60 transfers (∼275 generations).

### Short-term experiment: 7-day analysis of enrichment line 2B at transfer 70.

To examine the interplay between microbial community dynamics and cellulose degradation over the course of one transfer, an additional 30 filter paper tubes were inoculated during the passage of enrichment line 2B for transfer 70. We chose this line as the 2B community was stably highly cellulolytic, and we chose transfer 70 as it was the current transfer at the time of this short-term experiment. To analyze both variation between replicates and changes over short time periods, six tubes each were collected after 1, 48, and 168 h, and three tubes each were collected after 8, 24, 72, and 120 h. Samples, including the inoculum, were frozen for DNA extraction and sequencing, as detailed below. Twenty-one tubes were also inoculated to quantify cellulose degradation (as detailed below), and for this analysis, three experimental and four control (uninoculated) tubes were collected at each time point.

### Quantification of cellulose degradation.

For quantification analysis, during the transfer procedure, 200 μl of each community was inoculated into tubes containing 8 mL of M63 minimal medium and two preweighed 1- by 4-cm strips of cellulose filter paper (∼70 mg) in triplicate. Quantification of cellulose degradation of long-term enrichment line communities was performed every 5 to 10 transfers, and cultures were grown shaking for 10 days. Quantification of cellulose degradation in the short-term experiment was performed at each time point. For each analysis, four uninoculated tubes were used as controls. Filter paper degradation was quantified using a previously published acid-detergent method (60). Briefly, 16 mL of acid detergent was added to each tube, tubes were crimp sealed, and samples were autoclaved for 45 min at 121°C to separate all bacteria from the insoluble cellulose (61). Then, samples were immediately vacuum filtered through preweighed glass microfiber filters (Whatman GF/D, 2.7-μm pore size; GE Healthcare Life Sciences, Pittsburgh, PA), filters were washed with at least 200 mL hot deionized water (diH_2_O), dried overnight in a 105°C drying oven, and immediately reweighed to determine net cellulose loss.

### Collection of cultures for analyses.

In both the long-term and short-term experiments, at each transfer or time point, 20% glycerol freezer stocks were stored at −80°C. Also, samples were collected for DNA analysis by centrifuging 2 to 3 mL of culture for 12 min at 16,100 × *g* in a benchtop centrifuge, removing the supernatant, and storing cell pellets at −20°C. In the long-term experiments, at transfer 14 for enrichment lines 1A and 1B and transfer 20 for lines 2A, 2B, 3A, and 3B, 50-mL cultures were also inoculated to obtain enough material for both metagenome and metatranscriptome sequencing (Fig. 1F; see also Supplementary Methods at https://doi.org/10.6084/m9.figshare.12967751).

### DNA extraction and sequencing.

For the long-term enrichment experiment, DNA for 16S rRNA gene amplicon sequencing was extracted from transfers 0, 1, 5, 10, and 14 for enrichment line 1A and from transfers 0, 1, 5, 10, 14, 20, 30, 40, 50, and 60 for enrichment lines 2B and 3A. DNA was extracted for metagenomic sequencing at transfer 14 for enrichment lines 1A and 1B, and transfer 20 for enrichment lines 2A, 2B, 3A, and 3B using samples from 50-mL cultures (Fig. 1F). For the short-term experiment, DNA was extracted for 16S rRNA gene amplicon sequencing at all time points.

Extraction was performed following chemical and enzymatic lysis using a phenol/chloroform protocol (see Supplementary Methods at https://doi.org/10.6084/m9.figshare.12967751). For 16S rRNA gene-based community profiling, the V6 to V8 regions of the 16S rRNA gene were amplified using bacteria-specific primers and purified according to previously published protocols (30). DNA was sequenced using a 454 GS Junior sequencer with FLX Titanium chemistry and long-read modifications (62). For metagenomic sequencing, we confirmed DNA was not sheared by analyzing 200 ng using gel electrophoresis. Genomic DNA libraries were prepared, indexed, and sequenced together on one chip on an Illumina MiSeq sequencer with 2 × 300 bp read chemistry by the University of Wisconsin—Madison Biotechnology Center.

### RNA extraction and sequencing.

Total community RNA was extracted from single 50-mL cultures of enrichment line 1A at transfer 14 and lines 2B and 3A at transfer 20 using previously published protocols (60). These larger cultures ensured enough material to harvest both DNA (above) and RNA. The Ribo-Zero Gold rRNA removal kit (Epidemiology) was used to remove rRNA (Epicenter, Madison, WI). cDNA libraries were constructed, and Illumina HiSeq2500 Rapid Run 2 × 150 bp technology was used to sequence all three samples on one lane at the University of Wisconsin—Madison Biotechnology Center.

### 16S rRNA gene sequencing analysis.

Reads were analyzed using mothur version 1.35.1 (63). The data for enrichment lines across transfers (long-term experiment) were analyzed separately from the data for changes in enrichment line 2B transfer 70 across 7 days (short-term experiment). For each data set, reads were filtered to remove those without exact matches to barcode or primers, denoised, and trimmed to a maximum length of 200 bp. Reads were aligned to the Silva database v119 using the default kmer-based search methods, and reads were removed that did not overlap the region of interest (64). In the long-term experiment data set, 641 potential chimeras out of 3,169 unique sequences were identified using default settings in UCHIME v4.2.40 and removed in mothur (64, 65); in the short-term experiment data set, 610 potential chimeras out of 2,406 unique sequences were removed. Sequences were classified using a mothur-formatted version of Ribosomal Database Project (RDP) training set 9 with a cutoff 60% identity (66). All OTUs were constructed at a 97% similarity threshold. OTUs from the short-term experiment are differentiated with an “S” (e.g., “OTUS1”). Alpha and beta diversity analyses were performed in mothur using data subsampled to 1,018 reads per sample in the long-term experiment and 1,435 reads in the short-term experiment. The statistical analyses of alpha diversity indices and OTU abundance with cellulolytic ability and time were performed in JMP Pro 11 (SAS, Cary, NC). For the long-term experiment, the Spearman correlation of cellulolytic ability with the NMDS axes was determined using the corr.axis command in mothur. OTU clustering, cooccurrence analyses, and analysis of the *Cellvibrio* and *Cellulomonas* populations are detailed in the Supplementary Methods at https://doi.org/10.6084/m9.figshare.12967751.

### Metagenome assembly.

The metAMOS pipeline was used to remove adapters and trim reads using ea-utils, to assemble reads using IDBA-UD, and to call open reading frames using MetaGeneMark (67–71). Each sample was assembled independently. To assign taxonomy, scaffolds were analyzed using PhymmBL, v4.0 (72, 73). Because many of our scaffolds were relatively long, we only used BLAST results from PhymmBL. Taxonomic assignments with a BLAST score <1e^−100^ were retained.

### Assembly and analysis of MAGs.

MAGs were constructed using VizBin (74) clustering of assembled contigs from each metagenome individually; default settings were used, except the minimum contig length was 500 bp and contigs were annotated with their coverage. CheckM (75) was used to evaluate the completeness and contamination of each MAG. MAGs were discarded if they were less than 50% complete or more than 5% contaminated. The trimmed reads comprising each MAG were extracted using a custom python script, and these reads were reassembled using SPAdes with parameters –careful and –only-assembler (76). Quast v3.1 was used to compare original and reassembled genomes, and the version with a higher *N*_50_ was chosen for further analyses (77). Taxonomy of MAGs was assigned by annotating ribosomal proteins using phylosift v1.0.1 and viewed using Archaeopteryx v0.9901 beta (78, 79). Open reading frames were assigned using Prodigal (80).

### Metatranscriptome mapping.

Using Trimmomatic v0.32, we removed adapters from paired-end metatranscriptome reads using command ILLUMINACLIP:TruSeq3-PE-2.fa:2:30:10:1:true and trimmed reads using commands LEADING:3 TRAILING:3 MAXINFO:40:0.25 and MINLEN:70 (81). BWA was used to map trimmed reads to assembled metagenomes and to MAGs using default parameters (82). Then, samtools was used to produce a bam file and alignment statistics (83). Lastly, R packages Rsamtools, GenomicFeatures, and GenomicAlignments were used to count reads (84, 85).

### Annotation of metagenomes and MAGs.

The following hidden Markov models were used to annotate open reading frames: TIGRFAM, Pfam, resfam (antibiotic resistance), antiSMASH (secondary metabolism), NRPSsp (NRPS substrate), and dbCAN (carbohydrate active enzymes [CAZymes]) (86–91). Protein sequences were also assigned CAZyme annotations using a custom script that combines BLAST searching and Pfam searching against the CAZy database (59, 73, 92), KEGG annotations using BlastKOALA for MAGs and GhostKOALA for metagenomes searched against the genus_prokaryotes database (93), and SEED annotations using the desktop version of myRAST with default parameters (94). See additional details in the Supplementary Methods available at https://doi.org/10.6084/m9.figshare.12967751.

### *Cellvibrio* isolation and 16S rRNA gene sequencing.

To attempt to isolate cellulolytic community members, the freezer stock from the 3A enrichment line at transfer 50 was grown in an M63 filter paper test tube and dilution plated onto plates containing carbon-free M63 minimal medium with 1.5% agar (wt/vol) overlaid with CEL1 medium consisting of 1 g ammonium sulfate, 5 g Sigmacell-20 (crystalline cellulose; Sigma-Aldrich, St. Louis, MO), and 10 g of agar in 1 L, pH 7.2 (30). A colony capable of clearing the cellulose overlay was isolated onto 1.5% agar plates containing M63 minimal media with 5 g/L glucose and taxonomically classified as *Cellvibrio* using Sanger sequencing of the 16S rRNA gene (30).

### Data availability.

16S rRNA gene sequencing data, metagenomic data, and metatranscriptomic data are available at NCBI under BioProject number PRJNA691155 (SAMN17283214-9 and SAMN17375738-93).
